# Supplementary material for: Taylor’s law predicts unprecedented pulses of forest disturbance under global change
Source: Nat Commun. 2025 Jul 3;16:6133. doi: 10.1038/s41467-025-61585-5 (PMC12229331; doi:10.1038/s41467-025-61585-5)
Supplement: Supplementary file 1 — Supplementary Information [file 41467_2025_61585_MOESM1_ESM.pdf]

Supplementary information

Taylor's law predicts unprecedented pulses of forest disturbance under global change

Cornelius Senf[1,*], Rupert Seidl[1,2], Thomas Knoke[1] and Tommaso Jucker[3]

[1] Technical University of Munich, School of Life Sciences, Hans-Carl-von-Carlowitz-Platz 2, 85354 Freising, Germany

[2] Berchtesgaden National Park, Doktorberg 6, 83471 Berchtesgaden, Germany

[3] University of Bristol, School of Biological Sciences, Bristol BS8 1TQ, UK

* Corresponding author: cornelius.senf@tum.de

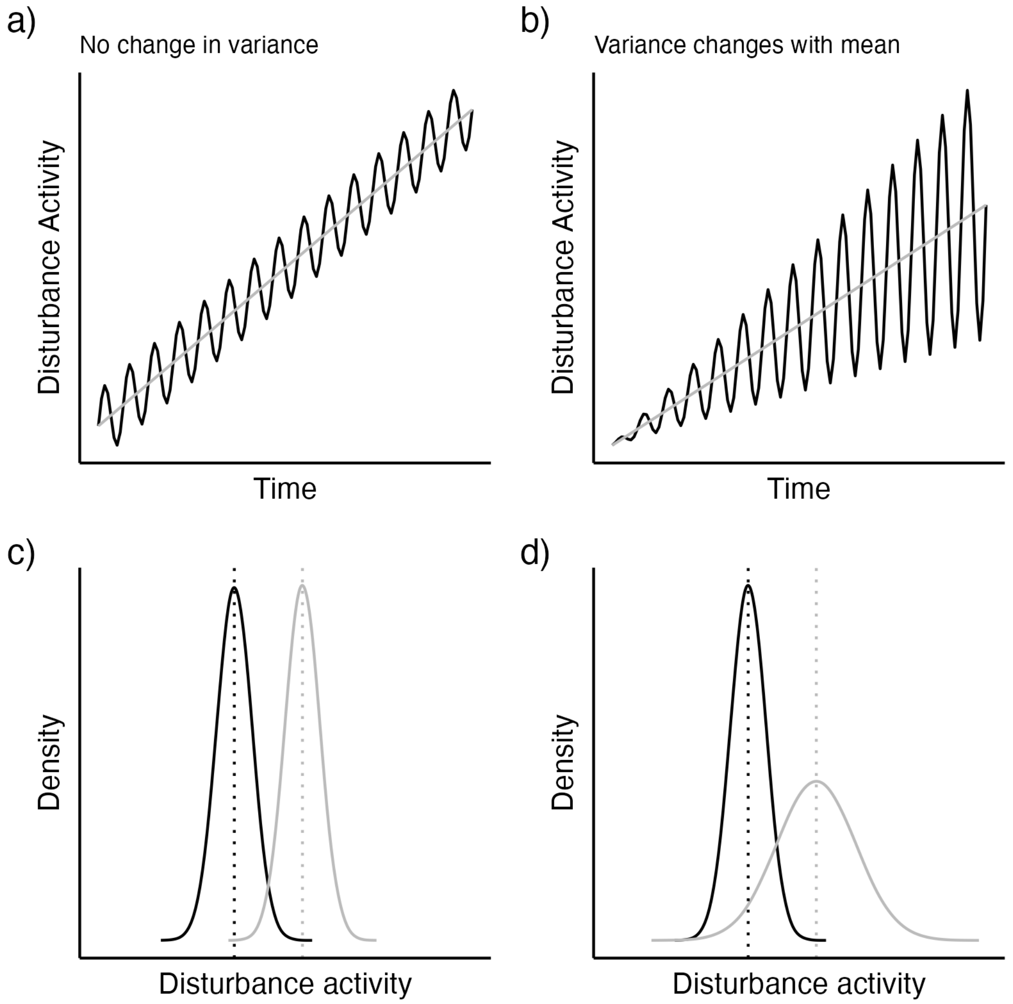

Supplementary Figure 1: Schematic diagram illustrating the implications of Taylor's law for forest disturbance dynamics. In the left column (a/c) variance does not increase with the mean for a hypothetical time series of annual disturbance activity (measured in terms of, e.g., disturbance rate, biomass loss), which would indicate no evidence of Taylor's law. The right column (b/d) instead shows an example in which the variance of the disturbance rate increases with the mean, as predicted by Taylor's law. The lower panels show how the distribution of disturbance rates, biomass loss, etc. changes when the mean disturbance rate is increased (from black to grey). In (a/c) the distribution remains unchanged, but in (b/d) as the mean increases the distribution widens. As a result, the likelihood of extreme disturbance values (defined as values far off the mean) also increases.

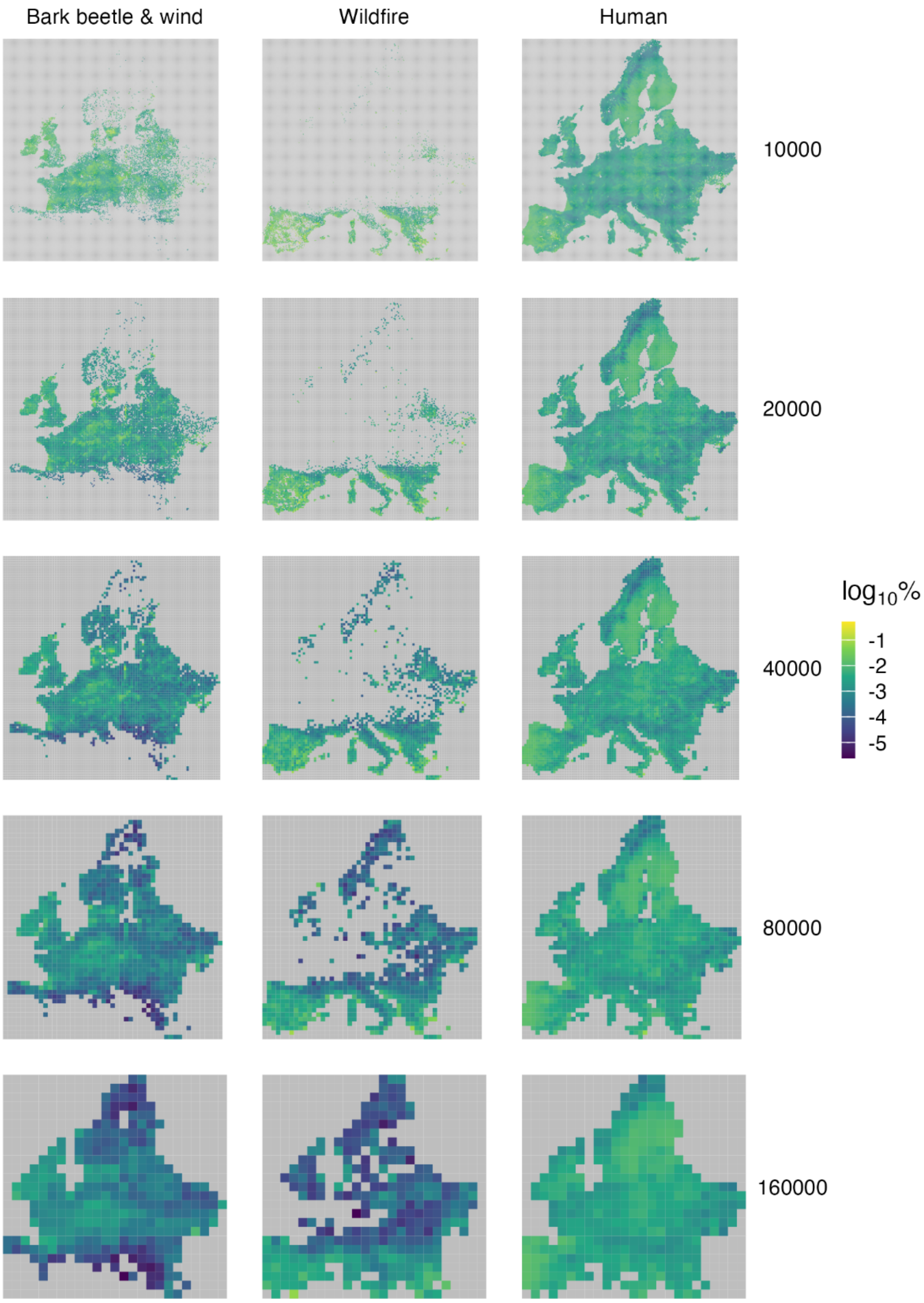

Supplementary Figure 2: Maps of mean disturbance rates log_{10}-transformed for better visibility and shown over all grains used in this study. Grey grid cells indicate no data.

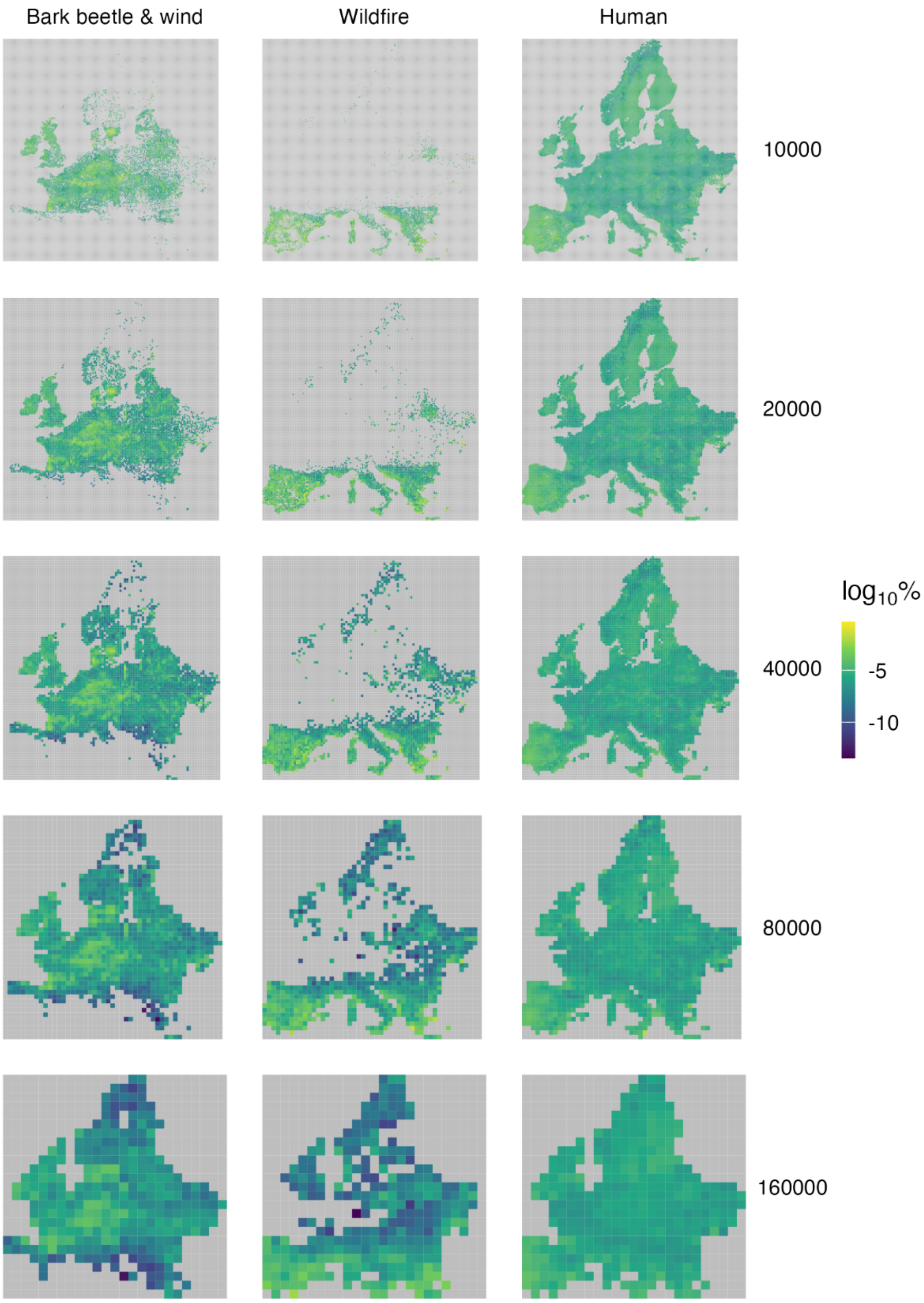

Supplementary Figure 3: Maps of variance in disturbance rates $\log_{10}$-transformed for better visibility and shown over all grains used in this study. Grey grid cells indicate no data.

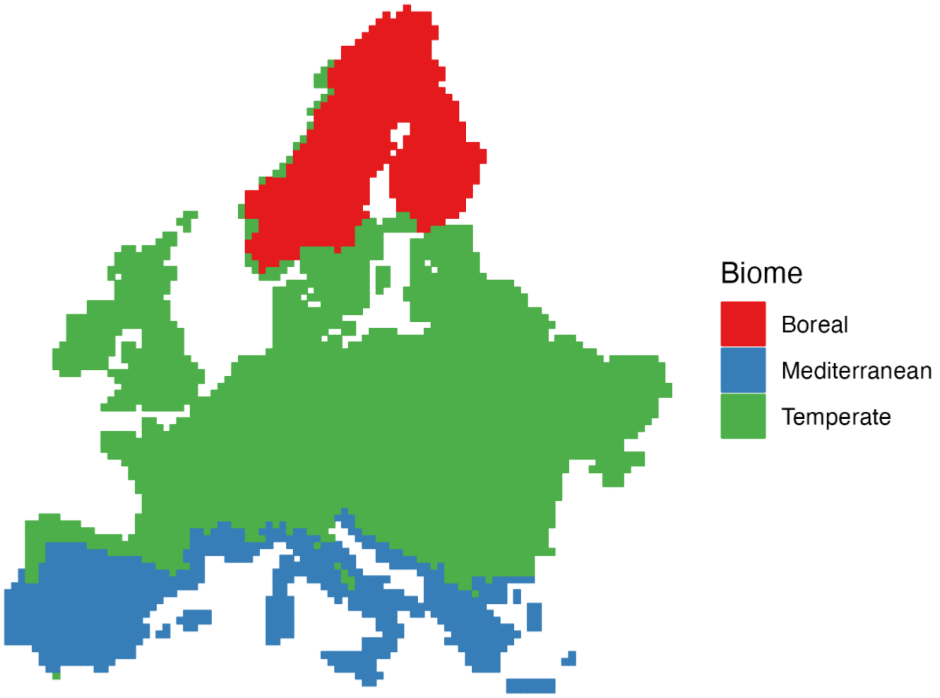

Supplementary Figure 4: Biome definition used in this study shown at a spatial resolution of 1,600 km^2.

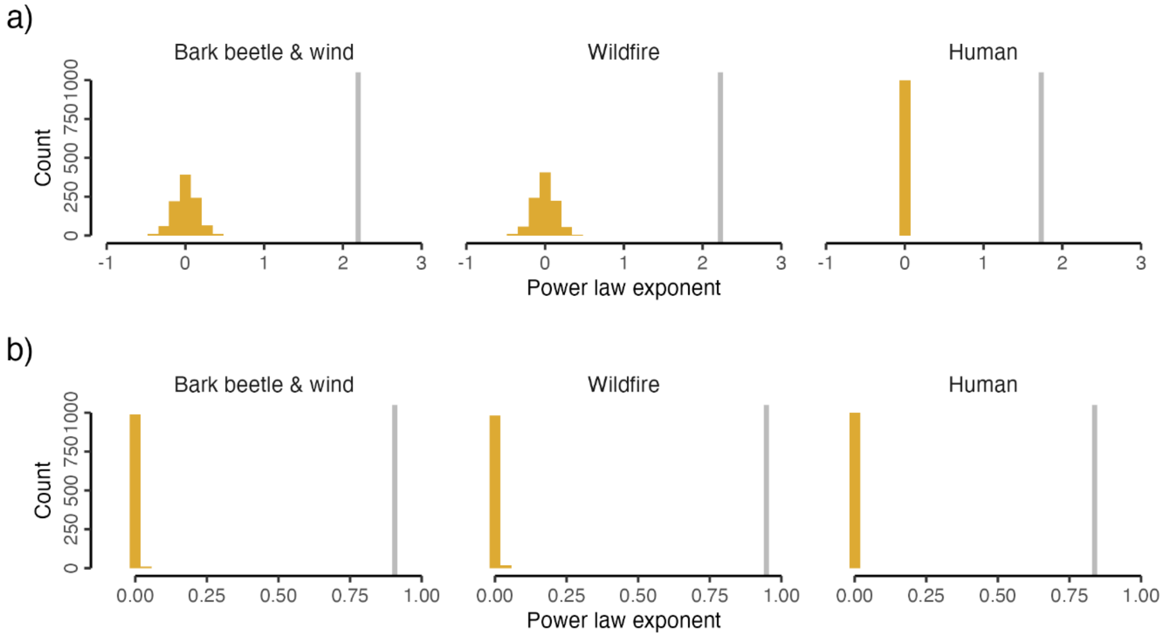

Supplementary figure 5: Comparison of estimated power law exponents (a; grey vertical line) and coefficient of determination (b; grey vertical line) to random draws from a null model that randomly reshuffles data points (yellow distribution). The random draws from the null model represent the null hypothesis (no scaling between mean and variance). The distinctly different exponents and models fits compared to the expectation under the null hypothesis provide strong evidence for a power law scaling between the mean and variance in disturbance rates.

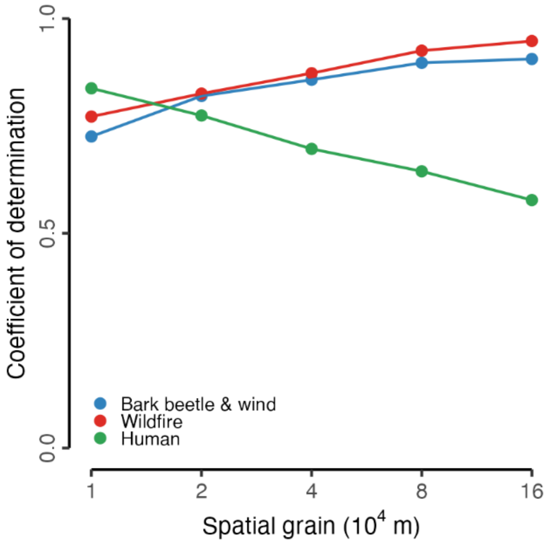

Supplementary figure 6: Coefficient of determination of the power law model linking temporal variability in disturbance rates to mean annual disturbance rate for the three disturbance agents over variable spatial grains.

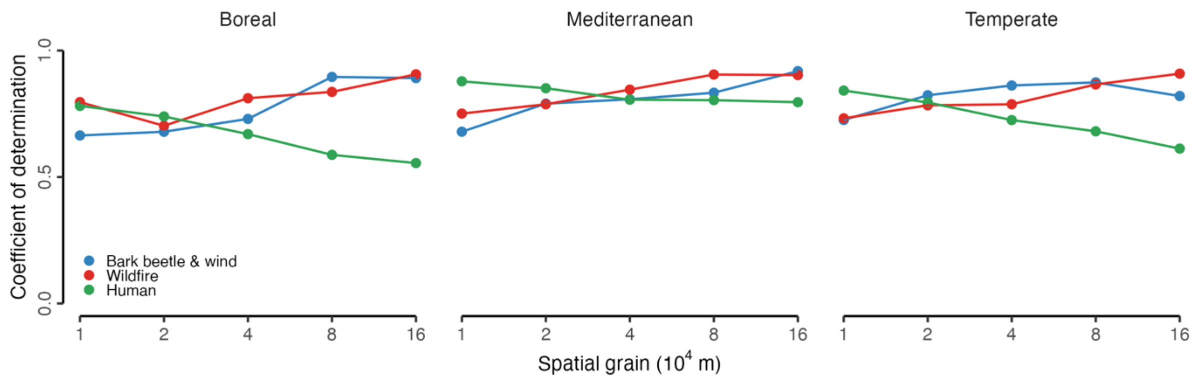

Supplementary figure 7: Coefficient of determination of the power law model linking temporal variability in disturbance rates to mean annual disturbance rate for the three disturbance agents over variable spatial grains within different biomes.

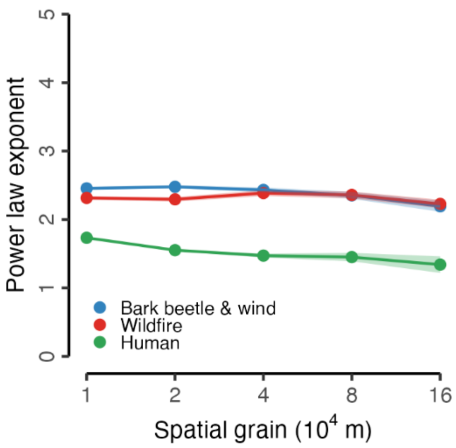

Supplementary figure 8: Power law exponent of the power law model linking temporal variability in disturbance rates to mean annual disturbance rate for the three disturbance agents over variable spatial grains. Ribbons indicate the 95% confidence interval.

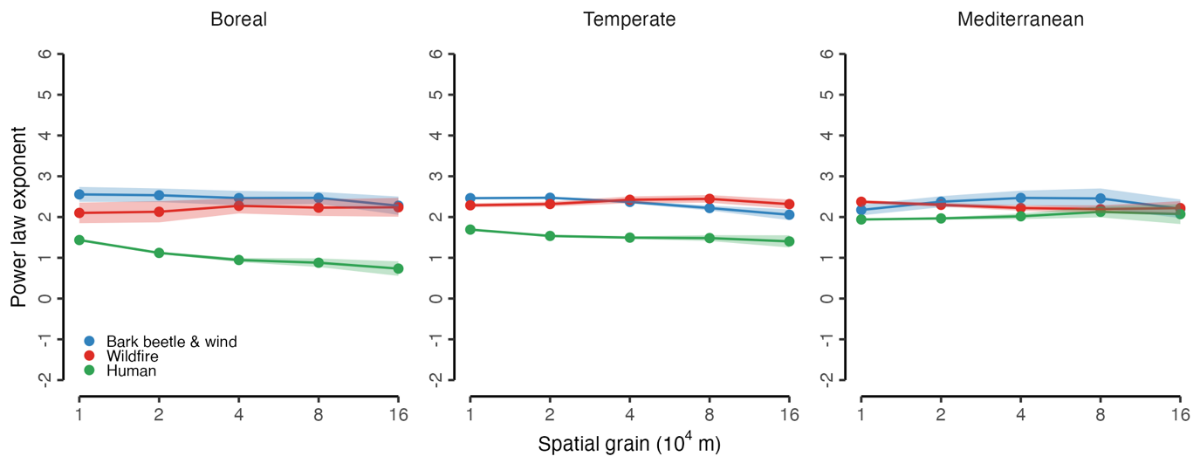

Supplementary figure 9: Power law exponent of the power law model linking temporal variability in disturbance rates to mean annual disturbance rate for the three disturbance agents over variable spatial grains within the three biomes. Ribbons indicate the 95% confidence interval.

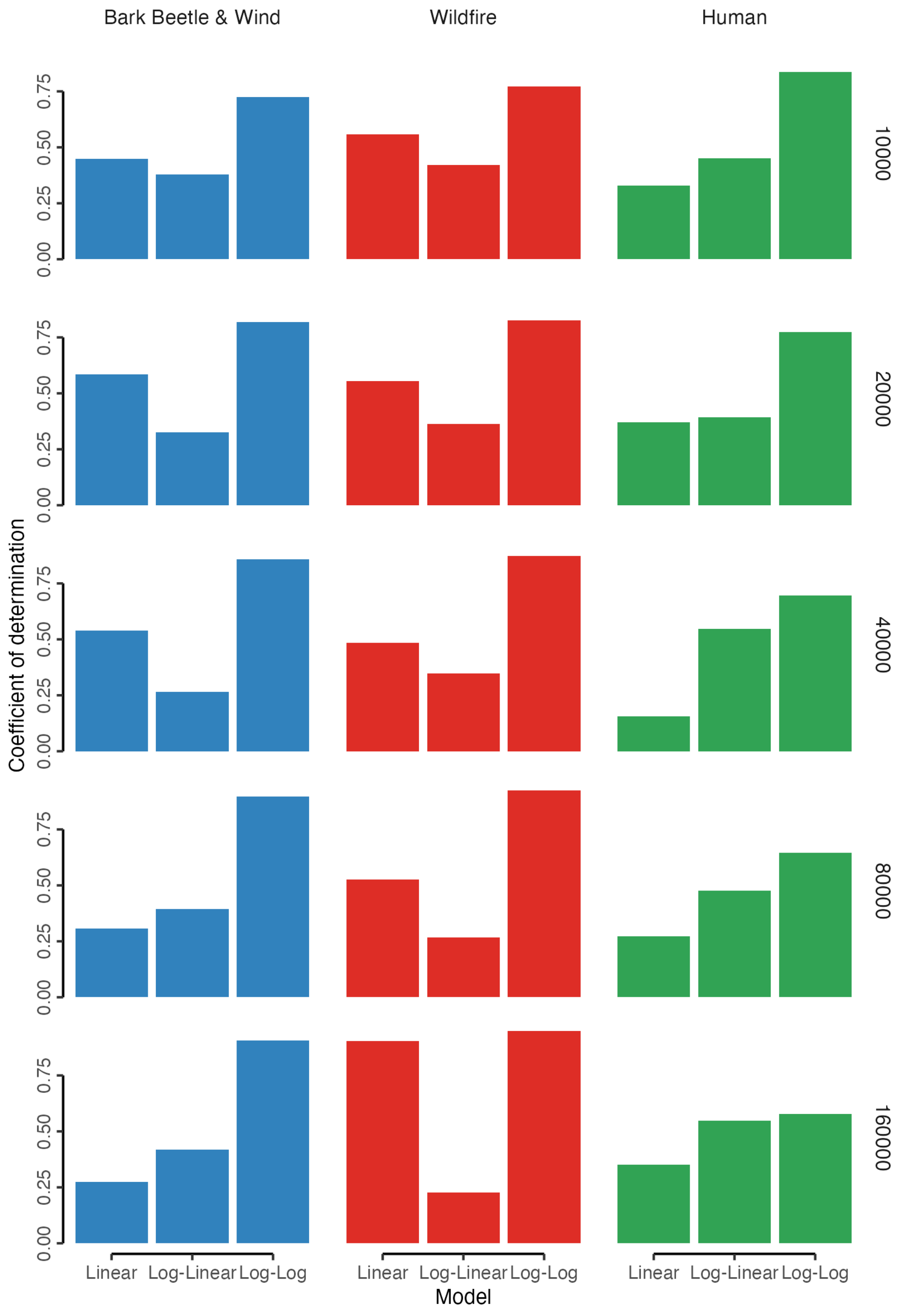

Supplementary figure 10: Comparison of different functional relationships to link mean and variance. We compared a linear model (both variance and mean on original scale), log-linear model (variance on $\log_{10}$-scale and mean on original scale), and a log-log model (i.e. our assumed Power-Law relationship with variance and mean on $\log_{10}$-scale). The coefficient of determination shows clear preference for the Power-Law model across agents and spatial grains.

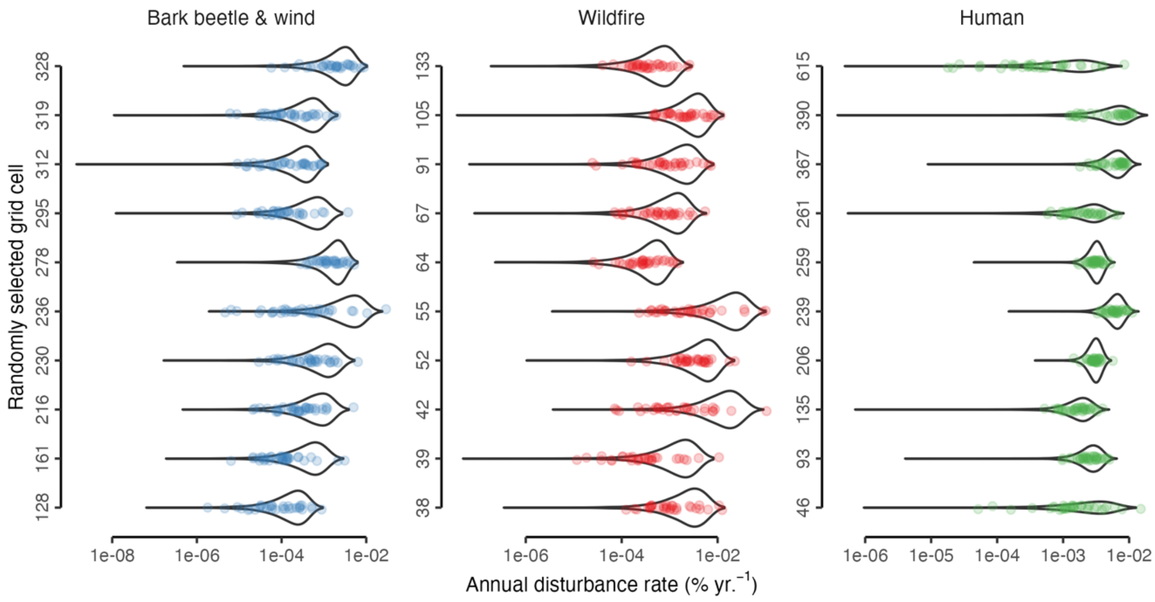

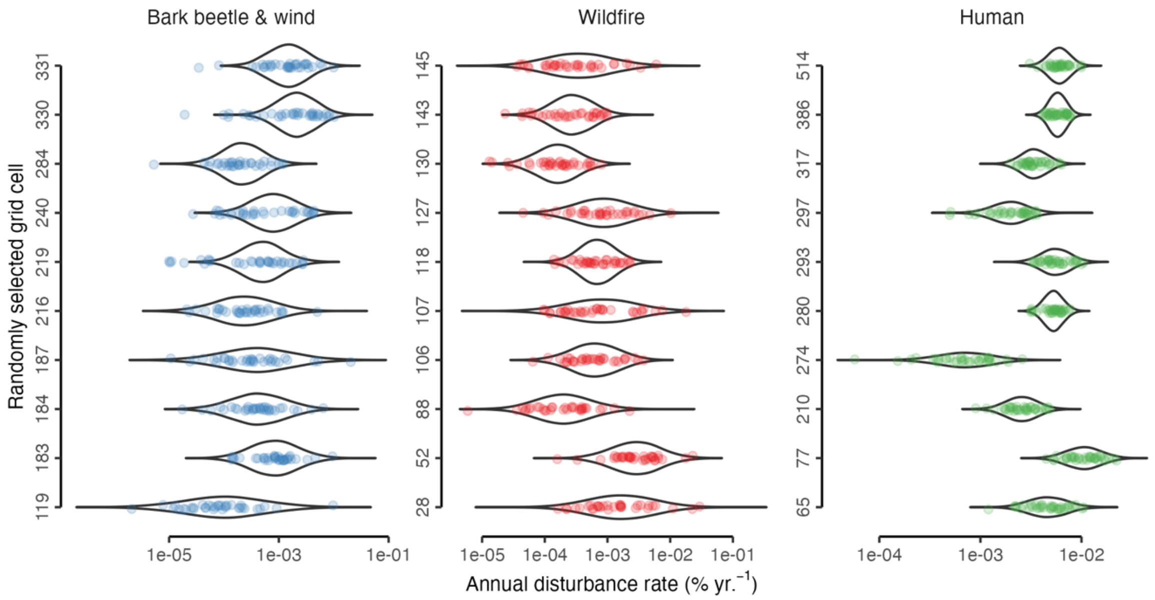

Supplementary figure 11: Test of the assumption of a squared-normal (upper plot) and log-normal (lower plot) distribution for simulating disturbance rates. The underlying violin plots show random draws from a squared-normal and log-normal distribution, respectively, for 10 randomly sampled grid cells per agent (at 25,600 km^2 resolution). The points show the observed disturbance rates of the respective grid cell. The log-normal distribution is better suited than the squared-normal distribution for reproducing observed disturbance rates, even at extreme values.
